# Bilateral thoracic Paravertebral block for immediate postoperative pain relief in the PACU: a prospective, observational study

**DOI:** 10.1186/s12871-017-0378-3

**Published:** 2017-07-05

**Authors:** Fei Liu, HuanKai Zhang, Yunxia Zuo

**Affiliations:** 1 Department of Anesthesiology, West China Hospital, Sichuan University, No37 Guoxue Street, Chengdu, Sichuan 610041 People’s Republic of China; 2 Department of Anesthesiology, Jie yang City people’s Hospital, Jie yang, Guangdong 522000 People’s Republic of China

**Keywords:** Thoracic paravertebral block, Postoperative pain, Post anesthesia care unit

## Abstract

**Background:**

To investigate the feasibility, effectiveness and safety of bilateral thoracic paravertebral block (TPVB) in the post anesthesia care unit (PACU) for pain relief in participants after laparotomy.

**Methods:**

A single shot of bilateral TPVB with 25 ml of 0.2% ropivacaine and 5 mg dexamethasone in combination for both sides at the 8th thoracic transverse level (T8) was performed on 201 participants who complained moderate to severe pain on arrival to PACU after laparotomy. The visual analog scale (VAS) pain scores at rest and on cough, heart rate, blood pressure, and pulse oximetry before and after bilateral TPVB for up to 1 h were recorded. The VAS Pain scores at rest and on cough at 24 h after bilateral TPVB were also recorded.

**Results:**

Bilateral TPVB was carried out successfully in all participants. The VAS pain scores at rest and on cough were 7.9 ± 1.6 and 8.7 ± 1.3 respectively pre-bilateral TPVB. The VAS pain scores at rest and on cough were significantly decreased to 1.1 ± 1.2 and 2.1 ± 1.6 respectively (*P* < 0.001) at 60 min after bilateral TPVB and to 2.1 ± 1.7 and 3.8 ± 1.9 at rest and on cough respectively ((*P* < 0.001) at 24 h after bilateral TPVB. At 10 min post-bilateral TPVB, only systolic blood pressure was reduced from 122 ± 19 mmHg to 111 ± 18 mmHg (*P* = 0.007) but then gradually became stable. No complications related to bilateral TPVB were observed.

**Conclusion:**

Bilateral TPVB can be provided for pain relief to the participants who suffer from moderate to severe pain after upper laparotomy in the PACU.

**Trial registration:**

Chinese Clinical Trial Registry: ChiCTR-ONN-16009229, Registered on 10 September 2016.

## Background

Pain following laparotomy is a common complaint in the PACU. Good pain management improves participant satisfaction and facilitates shorter PACU/hospital stay [1]. Although pain management in the PACU has been improving with opioid-free strategies, opioids are still commonly used of analgesia for surgical participants with moderate to severe pain [2]. Intravenous opioids can provide rapid and effective analgesia but their undesired side effects, including pruritus, nausea and vomiting, urinary retention, and respiratory depression, result in discomfort or even lethal consequences [3, 4].

TPVB is a technique of which local anesthetic is injected into the thoracic paravertebral space. The thoracic paravertebral space is a wedge-shaped space lying on either side of the vertebral column that fills with adipose tissue containing intercostal nerve, dorsal ramus, intercostal vessels, rami communicantes, and sympathetic trunk [5]. Therefore, TPVB in either side could produce ipsilateral, segmental, somatic and sympathetic nerve blockade in contiguous thoracic dermatomes [5, 6]. It has been demonstrated that preoperative TPVB provides an excellent intraoperative and postoperative analgesia with less adverse effects in thoracic and abdominal surgery [7–12]. However, the effectiveness of TPVB as a rescue technique in the PACU for acute postoperative pain relief remains unknown. In this study, participants in the PACU who suffer moderate to severe pain after upper laparotomy were treated with a single-injection of local anesthetic via bilateral TPVB. Its effectiveness and safety profile were assessed prospectively.

## Methods

The protocol was approved by the Ethics committee of West China Hospital, Sichuan University, China. Written informed consent was obtained from all participants prior to surgery. Participants were informed that they would be offered bilateral TPVB to be performed in the PACU if they reported moderate to severe pain which was not controlled adequately by the intraoperative and PACU analgesia that they had received. Inclusion criteria are: Age between 18 and 75 years; American Society of Anesthesiologists physical status 1 or 2; Laparotomy for hepatopancreatobiliary or gastrointestinal surgery; and participants with VAS pain score at rest ≥5 in the PACU. Exclusion criteria: allergy to local anesthetics; spinal deformity; coagulation disorders; local infection at the injection site. Accordingly, total 359 participants were recruited into this study.

**Fig. 1:**
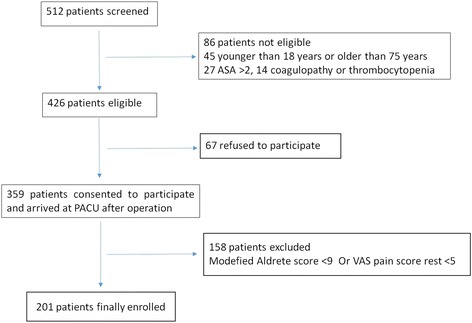
Participant recruitment flow chart

After arrived in the operation room, heart rate, non-invasive blood pressure, and pulse oximetry were monitored in all participants. General anesthesia was induced with intravenous midazolam 0.05 mg/kg, sufentanyl 0.3–0.5 μg/kg, propofol 2–3 mg/kg and cis-atracurium 0.2 mg/kg. Anesthesia was maintained with sevoflurane or propofol together with remifentanil 0.2–0.3 μg/kg/min. Analgesia consisted of Paricoxib 40 mg administered 30 min before incision, and tramadol 100 mg intravenously 30 min before the end of surgery. A patient control analgesia pump was started 30 min before the end of surgery, containing 1 μg/ml sufentanyl and 5 mg/ml tramadol, with a continuous infusion rate of 2 ml/h, 0.5 ml bolus dose and lockout interval set to 15 min. All participants were extubated at the end of surgery prior to transferring to the PACU. The VAS pain score at rest/cough and modified Aldrete score were assessed as soon as participants arrived in the PACU. Those 201 participants with the rest VAS ≥ 5 and modified Aldrete score ≥ 9 were finally enrolled in the study (Fig. 1).

**Fig. 2:**
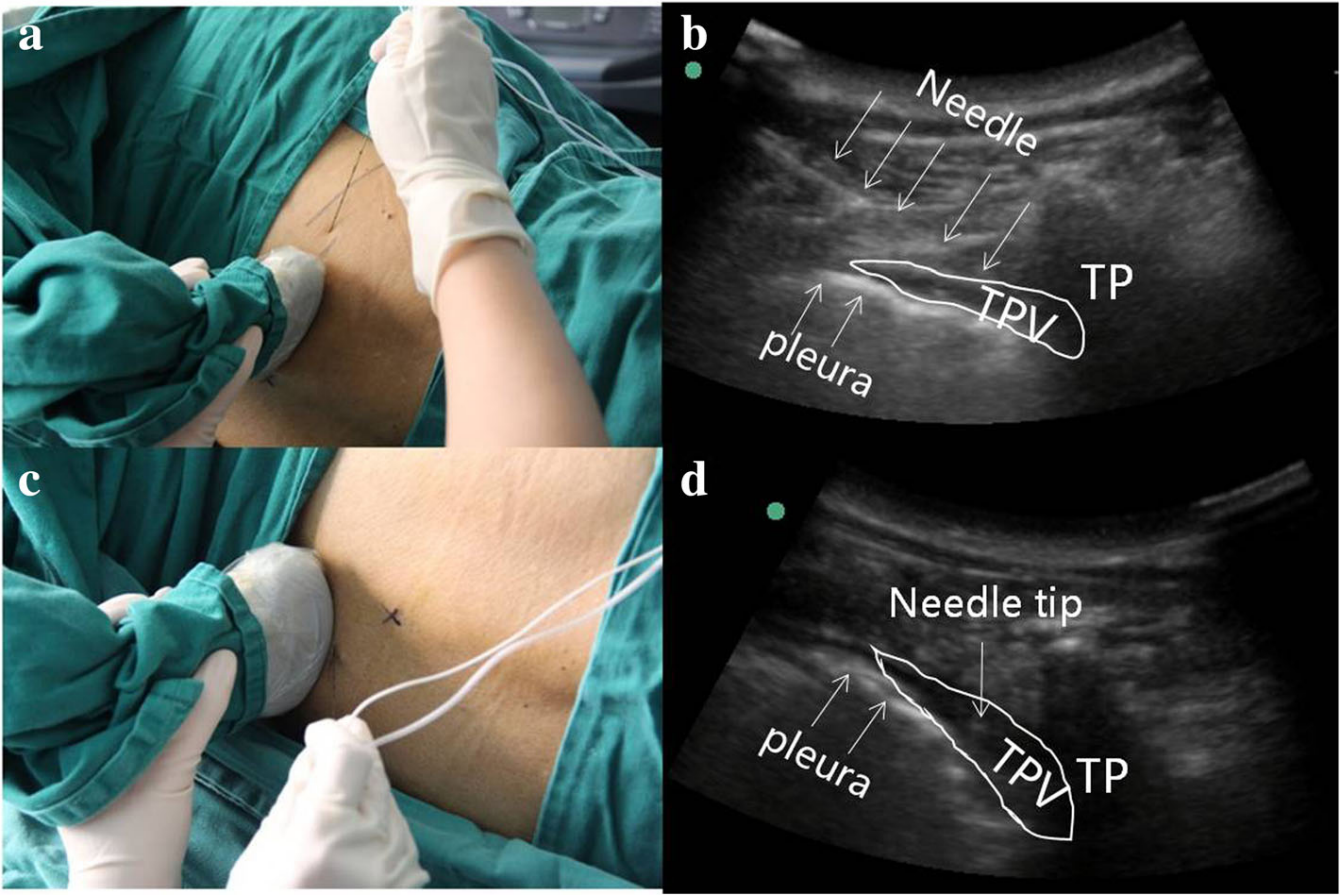
Ultrasound guided baliteral thoracic paravertebral block at T8 level. **a** and **b** illustrating the plane needle insertion to the right T8 paravertebral space. **c** and **d** illustrating the plane needle insertion to the left T8 paravertebral space. TP = Transverse process, TPV = Thoracic paravertebral space

Participants were placed in the left lateral decubitus position. A 2- to 5- MHz curved array transducer (M-Turbo, Sonosite, Bothell, USA) was used to scan from the midline laterally to identify the following anatomical landmarks: spinous process, transverse process, and the paravertebral space at the target vertebral level. The 8th thoracic transverse process was identified using ultrasound guidance from the 12th thoracic transverse process. An in-plane needle guidance technique with a 10 cm, 22 G insulated needle (PAJUNK GmbH Medizin technologie, Geisingen, Germany) was used to perform the right lateral TPVB under aseptic conditions. After perforating the costotransverse ligament, 0.2% ropivacaine 25 ml mixed with 5 mg dexamethasone was injected after confirming a negative aspiration for blood. Anterior movement of the pleura indicated the appropriate spread of the local anaesthetic mixture in the paravertebral space (Fig. 2a, b). An out plane needle guidance technique was performed on the contralateral side using the same drug combination (Fig. 2c, d).

The VAS pain score at rest and on cough, heart rate, blood pressure, and pulse oximetry before and after bilateral TPVB for up to 1 h were recorded by every 10 min. Hypotension was defined as a systolic blood pressure < 90 mmHg within 1 h after bilateral TPVB. The VAS pain score at rest and on cough 24 h after bilateral TPVB were also recorded.

Statistical analyses were performed with SAS for (Windows, version 9.13). The quantitative data were presented as mean ± SD. The categorical data were presented as frequency and/or percentage. Binary logistic regression and multivariate logistic regression models were used to identify risk factors of hypotension after bilateral TPVB.

## Results

Effective bilateral TPVB was achieved in 139 male and 62 female participants with age 52.8 ± 12.1 years and BMI 22.0 ± 2.8 kg/m2. The length of surgery was 178.9 ± 62.7 min. No complications associated with the bilateral TPVB (pneumothorax, pleural puncture, nerve injury, or vascular puncture) were observed in this study.

**Fig. 3:**
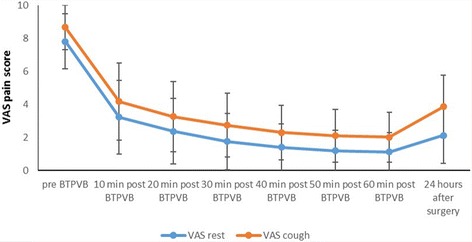
VAS pain scores at rest and cough before and after bilateral TPVB

The VAS scores at rest and on cough were rapidly reduced from 7.9 ± 1.6 and 8.7 ± 1.3 of baseline before bilateral TPVB to 3.3 ± 2.2 and 4.2 ± 2.3 10 min after bilateral TPVB respectively (*P* < 0.001) and then gradually decreased to 1.1 ± 1.2 and 2.1 ± 1.6 respectively (*P* < 0.001) at 1 h after injection (Fig. 3). No rescue analgesic was needed after bilateral TPVB in those patients after bilateral TPVB during their PACU stay. The VAS scores were 2.1 ± 1.6 and 3.8 ± 1.9 at rest and on cough 24 h after bilateral TPVB.

**Fig. 4:**
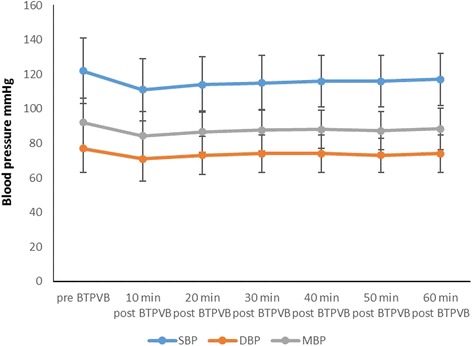
The changes of systolic blood pressure, diastolic blood pressure and mean blood pressure before and post bilateral TPVB

**Fig. 5:**
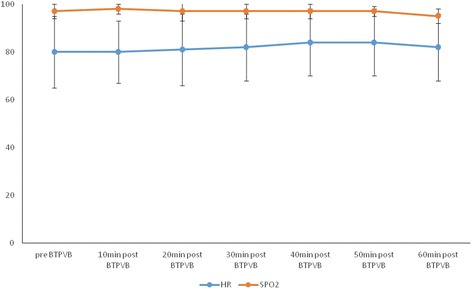
Heart rate and SPO_2_ before and after bilateral TPVB

Systolic blood pressure was significantly decreased to 111 ± 18 mmHg (*P* < 0.001) at 10 min after bilateral TPVB from the baseline of 123 ± 19 mmHg. By definition, hypotension (systolic blood pressure < 90 mmHg) occurred in 24 patients (11.9%). However, they were closely monitored and their systolic blood pressure were gradually returned to the baseline within 60 min after bilateral TPVB (Fig. 4). With a binary logistic regression model analysis, body weight, operative time, systolic blood pressure, diastolic blood pressure and mean blood pressure lower readings before bilateral TPVB were risk factors for hypotension (Table 1). Using multivariate logistic regression model analysis, low systolic blood pressure before bilateral TPVB and heavy body weight were indentified to be independent risk factors for hypotension after bilateral TPVB (Table 2). Diastolic blood pressure was to be a similar pattern trend change as systolic blood pressure (Fig. 4). No significant changes of SPO2 and heart rate were found throughout the study (Fig. 5).

**Table 1:** Binary logistic regression model for risk factors of hypotension after bilateral TPVB

Variable	Hypotensive group 24	Non-Hypotensive group 177	P	OR
Sex(male/female)	18/6	120/57	0.477	1.425
Age	51 ± 11	53 ± 12	0.465	0.987
Body weight	63.9 ± 8.8	58.8 ± 9.4	0.015*	1.059
Height	166.2 ± 6.8	164.1 ± 7.4	0.202	1.039
BMI	22.9 ± 2.6	21.8 ± 2.8	0.053	1.170
Operative time	205 ± 49	174 ± 66	0.030*	1.007
Heart rate	82 ± 14	80 ± 14	0.674	1.007
Systolic blood pressure	107 ± 16	125 ± 19	<0.001*	0.938
Diastolic blood pressure	71 ± 15	78 ± 13	0.011*	0.957
Mean blood pressure	83 ± 14	94 ± 14	0.001*	0.938
VAS pain score rest	7.6 ± 1.9	7.8 ± 1.6	0.618	0.937
VAS pain score cough	8.4 ± 1.6	8.7 ± 1.3	0.381	0.873

*BMI* Body Mass Index, *VAS* Visual Analog Scale,* *P* < 0.05

**Table 2:** Multivariate logistic regression model for risk factors of hypotension

Variable	Estimate	StdErr	Wald	P	OR
BW	0.066	0.026	6.357	0.012	1.068
Systolic blood pressure	−0.066	0.017	15.567	<0.001	0.936

*BW* Body Weight

## Discussion

Our results showed that ultrasound-guided bilateral TPVB is an effective rescue analgesic technique for moderate and severe pain which is not adequately controlled by conventional intravenous analgesia after upper laparotomy.

It has been reported that in thoracic surgeries, unilateral TPVB was as effective as epidural analgesia but with much less side effects [13, 14]. Furthermore, considerable evidence showed that TPVB in addition to GA provide a better postoperative pain control with fewer adverse effects when compared GA alone in video-assisted thoracoscopic surgery and breast surgery [15–17]. However, it was not often reported to be used for postoperative pain relief after laparotomy under GA. Richardson et al. have reviewed 541 participants undergoing bilateral TPVB in 12 various studies, and they concluded that bilateral TPVB is safe and effective for thoracic and abdominal surgery [12]. However, the methods and outcomes in those studies are variable. Although many studies found that unilateral TPVB produced similar analgesia efficacy as compared with epidural block for thoracic surgery, a few studies have yet been compared bilateral TPVB with epidural analgesia for laparotomy which is generally considered to be a standard approach for postoperative pain relief after laparotomy. Recently Schreiber et al. found that there was modest analgesic advantage of thoracic epidural over bilateral TPVB for participants after open liver resection [18]. However, as the author also pointed out, technical risk and rare but serious complications such as epidural hematoma, perioperative hypotension related to epidural block can not be ignored. Furthermore, Schreiber et al. used a traditional landmark-based method for TPVB catheter insertion instead of ultrasound guidance or intraoperative placement by surgeons under direct vision, which may lead to more variable analgesic efficacy.

Instead of multiple injection at different thoracic level, a single injection of thoracic paravertebral block on each side at the T8 level was performed to reduce procedure time in a cohort of participants who were already in considerable pain. A previous study investigated the spread of local anesthetic in cadavers, and found no difference in paravertebral segment spread over 3–4 vertebral segments between a single versus dual-injection technique [19]. Our study showed that a single injection with large volume can provide good analgesic effect for large middle abdominal incision, indicating that multi-injections are not necessary.

Dynamic VAS scores at rest and on cough showed that bilateral TPVB provided effective analgesia with a rapid onset, supporting the use of bilateral TPVB in the PACU as an effective rescue analgesic technique. The rapid onset could be explained by the spinal nerve in the thoracic paravertebral space lacking both an epineurium and part of the perineurium, and with only a thin membranous root sheath. All these could enhance rapid local anaesthetic penetration, providing effect and rapid analgesia [20]. A mild decrease of systolic pressure without further treatment at 10 min after block and no changes of heart rate and SPO_2_ indicated that bilateral TPVB has minimal inhibitory effects on cardiorespiratory system.

Our study is limited as we assessed the efficacy of bilateral TPVB as a rescue technique for inadequate analgesia from our standard intravenous regimen, rather than comparing the efficacy of bilateral TPVB prior to surgery. A different study is required to test that research question. Furthermore, our study is observational without epidural block and or intravenous analgesic as the control groups and therefore we couldn’t conclude if bilateral TPVB gives better pain relief and has less side effects. Lastly, only a single shot was conducted and therefore, it is not known how the picture of bilateral TPVB with continuous infusions by catheter insertion is when compared with a single shot injection. Anyhow, our study showed that bilateral TPVB under the aid of ultrasound guide at the T8 level in the PACU provides immediate and good analgesia for patients suffering moderate or severe pain after laparotomy without significant side effects. However, due to the nature of the observatory study reported here, clinical trials are needed to further confirm its clinical safety and effectiveness.

## Conclusions

In summary, the use of ultrasound guided bilateral TPVB as a rescue analgesic technique in the PACU is effective and provides rapid onset of analgesia.

## Abbreviations

PACU: Post anesthesia care unit
T8: 8th thoracic transverse level
TPVB: Thoracic paravertebral block
VAS: Visual analog scale.
